# Tuberculose surrénalienne bilatérale: à propos d’un cas

**DOI:** 10.11604/pamj.2018.29.212.15459

**Published:** 2018-04-13

**Authors:** Nawal Bouknani, Daoud Bentaleb, Hasna Belgadir, Omar Amriss, Nadia Moussali, Naima Elbenna

**Affiliations:** 1 Service de Radiologie 20 Aout 1953, CHU Ibn Rochd de Casablanca, Casablanca, Maroc

**Keywords:** Tuberculose surrénalienne, incidentalome, imagerie, TDM, Adrenal tuberculosis, incidentaloma, imaging, CT scan

## Abstract

La localisation surrénalienne isolée de la tuberculose représente moins de 2% des incidentalomes surrénaliens. C'est la cause infectieuse la plus fréquente des insuffisances surrénaliennes. Nous rapportons le cas d'un patient âgé de 53 ans, sans antécédents particuliers, qui présente un tableau d'insuffisance surrénalienne lente évoluant depuis six mois. L'examen physique n'a pas retrouvé de masse ni d'hépato-splénomégalie. La tension artérielle était à 120/60 mmHg. L'examen biologique n'a pas objectivé de syndrome inflammatoire et un taux de LDH normal. La TDM a objectivé une hypertrophie bilatérale des glandes surrénales siège de calcifications. L'intradermo réaction à la tuberculine était positive à 25mm. La recherche de BK dans les expectorations et dans les urines était négative. Le test au Quantiferon^®^ était positif. Un traitement antibacillaire d'épreuve a été démarré avec une amélioration clinique avec prise de poids de 5kg en 12 mois. Les dosages hormonaux restent bas.

## Abstract (en)

Isolated adrenal tuberculosis accounts for less than 2% of adrenal incidentalomas. This is the most frequent infectious cause of adrenal insufficiencies. We report the case of a 53-year old patient with no previous medical history presenting with adrenal insufficiency with slow progression over six months. Physical examination didn’t show any mass or hepatosplenomegaly. Blood pressure was 120/60 mmHg. Laboratory tests didn’t show inflammatory syndrome. LDH level was normal. CT scan showed bilateral hypertrophy of the adrenal glands characterized by calcifications. Intradermo tuberculin reaction was positive at 25mm. The analyses to detect Koch’s bacillus in the sputum and in the urine were negative. Quantiferon^®^ test was positive. Trial antibacillar treatment was started with clinical improvement and 5kg weight gain in 12 months. Hormonal assays were low.

## Introduction

La localisation surrénalienne isolée de la tuberculose est rare et représente moins de 2% des étiologies des Incidentalomes surrénaliens. C'est la cause infectieuse la plus fréquente des insuffisances surrénaliennes. A défaut d'un contexte évocateur où le diagnostic est facile, une preuve histologique est nécessaire.

## Patient et observation

**Figure 1:**
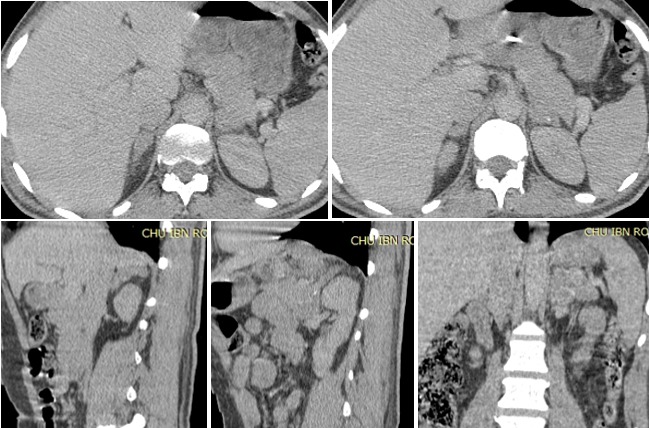
TDM surrénalienne en coupes axiales, sagittales et coronale montrent une hypertropie bilatérale des glandes surrénales plus marquée à gauche avec des calcifications

Il s'agit d'un patient âgé de 53 ans, sans antécédent particulier, qui présente un tableau d'insuffisance surrénalienne lente évoluant depuis six mois, avec un taux de cortisolémie bas à 45,26mmoL/L. L'examen physique n'a pas retrouvé de masse ni d'hépato-splénomégalie. La tension artérielle était à 120/60 mmHg. L'examen biologique n'a pas objectivé de syndrome inflammatoire et un taux de LDH normal. Une échographie puis un scanner ont objectivé une hypertrophie bilatérale des glandes surrénales plus marquée à gauche siège de calcifications (Figure 1). Une radiographie thoracique était normale. L'intradermo réaction à la tuberculine était positive à 25mm. La recherche de BK dans les expectorations et dans les urines était négative. Le test au Quantiferon^®^ était positif. Un traitement antibacillaire d'épreuve a été démarré associé à un traitement hormonal substitutif avec une amélioration clinique avec prise de poids de 5kg en 12 mois. Les dosages hormonaux restent bas.

## Discussion

Décrite pour la première fois par Addison en 1855, la tuberculose surrénalienne est une cause rare des masses surrénaliennes (moins de 2%) [1]. Dans une série autopsique chez des patients atteint de tuberculose active, la localisation surrénalienne a représenté 6% [2]. Dans une série de 238 incidentalome [3] la tuberculose représente uniquement 1,3%. La tuberculose se propage par voie hématogène aux glandes surrénaliennes. Elle est le plus souvent secondaire à une tuberculose génito-urinaire non ou mal traitée, ou plus rarement primitive due à une réactivation de la maladie [4]. Les signes cliniques de l'atteinte isolée de la surrénale ne sont pas spécifiques : signes généraux, douleurs ou sensation de pesanteur… Les signes d'insuffisance surrénalienne sont tardifs n'apparaissent qu'après destruction d'au moins 90% de la glande (phase chronique ou inactive) [5]. L'échographie objective une masse surrénalienne uni ou bilatérale. La TDM est plus sensible et permet une caractérisation meilleure. L'aspect radiologique dépend du stade évolutif de la maladie. Dans sa phase aiguë, la TDM montre une hypertrophie surrénalienne souvent bilatérale. L'injection de produit de contraste iodé peut objectiver une zone de nécrose centrale (nécrose caséeuse). La maladie dans cette phase est presque toujours asymptomatique (incidentalome). À la phase chronique, une atrophie, des calcifications sont fréquents [6]. L'aspect radiologique de la tuberculose surrénalienne n'est pas spécifique [7]. Plusieurs diagnostics (Tableau 1) peuvent être discutés en cas d'incidentalome surrénalien [8]. Il est impératif d'éliminer un phéochromocytome avant de procéder à tout geste diagnostique invasif. [3]. Le diagnostic de certitude repose sur la biopsie écho ou scannoguidée avec étude histologique qui objective un granulome épithélio-gigantocellulaire avec nécrose caséeuse spécifique de la tuberculose. La biopsie surrénale n'est pas nécessaire chez les patients atteints de tuberculose extra-surrénalienne [9]. Un traitement d'épreuve est instauré dans ces cas. Le bilan à la recherche d'autres localisations intéresse particulièrement les formes urogénitale, pulmonaire et osseuse : recherche de Bacilles de Koch dans les urines, dans les expectorations… Un traitement hormonal substitutif (glucocorticoïde et minéralocorticoïdes) est administré en cas d'insuffisance surrénalienne. Le traitement spécifique se base sur les antibacillaires (rifampicine; isoniazide; pyrazinamide; éthambutol) selon un régime établi. Le suivi repose essentiellement sur l'examen clinique (prise de poids, disparition des signes cliniques…). Un contrôle scannographique peut s'avérer nécessaire, notamment en cas de diagnostic à la phase aigüe de la maladie, pour évaluer la réduction de la masse surrénalienne.

**Tableau 1:** Etiologies des incidentalomes surrénaliens

**Sécrétant (≥ 15%)**
Adénome (aldostérone or cortisol)
Carcinome
Phéochromocytome
Hyperplasie nodulaire
Hyperplasie macro nodulaire massive
**Non sécrétant**
Adénome
Myélolipome
Neuroblastome
Ganglioneurome
Hémangiome
Carcinome
Métastase
Kyste
Hémorragie
Granulome
Amylose
Pathologie infiltrative

## Conclusion

La tuberculose surrénalienne est une cause rare des incidentalomes. La tomodensitométrie et la biopsie écho ou scannoguidée jouent un rôle primordial dans le diagnostic ainsi une prise en charge précoce. Le traitement repose sur les antibacillaires. Un traitement précoce et une bonne observance permettent d'améliorer le pronostic.

## Conflits d’intérêts

Les auteurs ne déclarent aucun conflit d'intérêts.
